# 4-[(2,5-Dimethyl-1,3-thia­zol-4-yl)meth­yl]-4-hydr­oxy-2-methyl­isoquinoline-1,3(2*H*,4*H*)-dione

**DOI:** 10.1107/S1600536810011141

**Published:** 2010-03-27

**Authors:** Hoong-Kun Fun, Jia Hao Goh, Haitao Yu, Yan Zhang

**Affiliations:** a X-ray Crystallography Unit, School of Physics, Universiti Sains Malaysia, 11800 USM, Penang, Malaysia; b School of Chemistry and Chemical Engineering, Nanjing University, Nanjing 210093, People’s Republic of China

## Abstract

In the title isoquinoline­dione compound, C_16_H_16_N_2_O_3_S, the piperidine ring in the tetra­hydro­isoquinoline ring system adopts a half-boat conformation. The essentially planar thia­zole ring [maximum deviation = 0.007 (2) Å] makes a dihedral angle of 34.49 (7)° with the mean plane through the tetra­hydro­isoquinoline ring system. In the crystal structure, two neighbouring mol­ecules are linked *via* pairs of O—H⋯N and C—H⋯O hydrogen bonds into inversion-related dimers incorporating *R*_2_^2^(9) hydrogen-bond ring motifs. These dimers are further linked by weak inter­molecular C—H⋯π inter­actions.

## Related literature

For general background to and applications of isoquinoline­dione derivatives, see: Griesbeck *et al.* (2003 ▶); Suau & Villatoro (1994 ▶); Zhang *et al.* (2000 ▶, 2004 ▶). For ring conformations, see: Cremer & Pople (1975 ▶). For graph-set descriptions of hydrogen-bond ring motifs, see: Bernstein *et al.* (1995 ▶). For related structures, see: Fun *et al.* (2010**a* ▶,*b* ▶,c* ▶); Wang *et al.* (2000 ▶). For the stability of the temperature controller used for the data collection, see: Cosier & Glazer (1986 ▶).
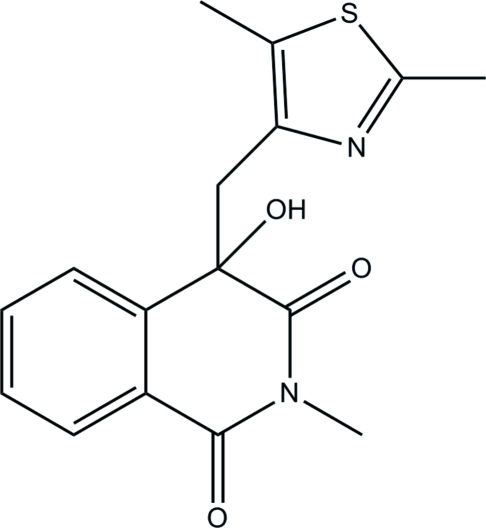


## Experimental

### 

#### Crystal data

C_16_H_16_N_2_O_3_S
*M* *_r_* = 316.37
Monoclinic, 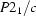

*a* = 8.5793 (2) Å
*b* = 10.4438 (2) Å
*c* = 17.5496 (3) Å
β = 114.304 (1)°
*V* = 1433.09 (5) Å^3^
*Z* = 4
Cu *K*α radiation
μ = 2.14 mm^−1^
*T* = 100 K
0.32 × 0.19 × 0.12 mm

#### Data collection

Bruker SMART APEX DUO CCD area-detector diffractometer
Absorption correction: multi-scan (*SADABS*; Bruker, 2009 ▶) *T*_min_ = 0.546, *T*_max_ = 0.782
22926 measured reflections
2343 independent reflections
2311 reflections with *I* > 2σ(*I*)
*R*_int_ = 0.021

#### Refinement

*R*[*F* ^2^ > 2σ(*F* ^2^)] = 0.041
*wR*(*F* ^2^) = 0.135
*S* = 1.33
2343 reflections
264 parameters
All H-atom parameters refined
Δρ_max_ = 0.87 e Å^−3^
Δρ_min_ = −1.01 e Å^−3^

### 

Data collection: *APEX2* (Bruker, 2009 ▶); cell refinement: *SAINT* (Bruker, 2009 ▶); data reduction: *SAINT*; program(s) used to solve structure: *SHELXTL* (Sheldrick, 2008 ▶); program(s) used to refine structure: *SHELXTL*; molecular graphics: *SHELXTL*; software used to prepare material for publication: *SHELXTL* and *PLATON* (Spek, 2009 ▶).

## Supplementary Material

Crystal structure: contains datablocks global, I. DOI: 10.1107/S1600536810011141/sj2758sup1.cif

Structure factors: contains datablocks I. DOI: 10.1107/S1600536810011141/sj2758Isup2.hkl

Additional supplementary materials:  crystallographic information; 3D view; checkCIF report

## Figures and Tables

**Table 1:** Hydrogen-bond geometry (Å, °) *Cg*1 and *Cg*2 are the centroids of the C3–C8 benzene ring and the C11/C12/S1/C13/N2 thia­zol ring, respectively.

*D*—H⋯*A*	*D*—H	H⋯*A*	*D*⋯*A*	*D*—H⋯*A*
O3—H1*O*3⋯N2^i^	0.84 (3)	2.35 (3)	3.174 (2)	167 (3)
C10—H10*A*⋯O1^i^	0.96 (2)	2.39 (2)	3.163 (2)	138 (2)
C14—H14*B*⋯*Cg*1^ii^	0.97 (3)	2.71 (3)	3.403 (2)	129 (2)
C15—H15*B*⋯*Cg*2^iii^	0.97 (3)	2.89 (2)	3.537 (2)	125.4 (18)

Symmetry codes: (i) 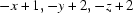; (ii) 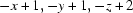; (iii) 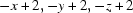.

**Fig. 1.**
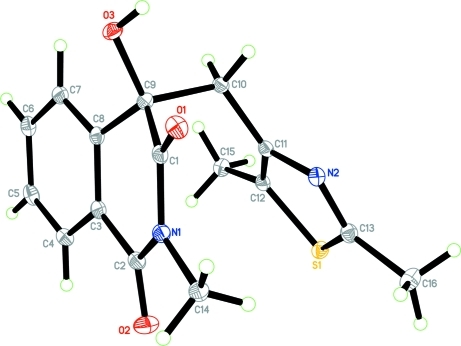
The asymmetric unit of the title compound, showing the atomic numbering scheme. Displacement ellipsoids are drawn at the 30% probability level.

**Fig. 2.**
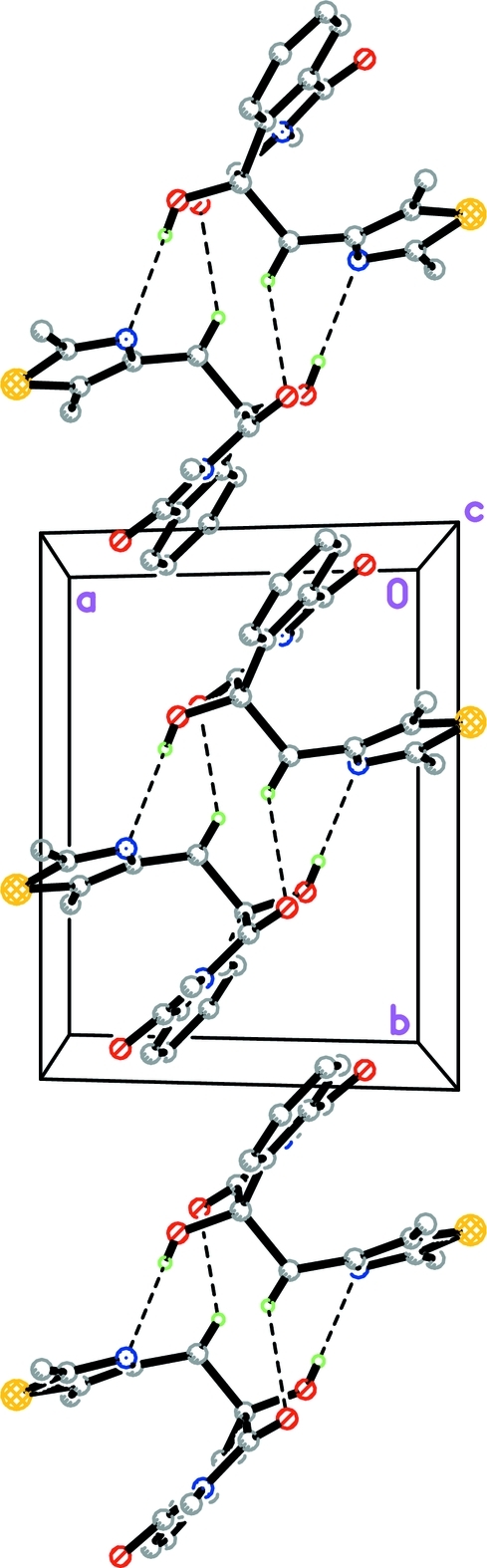
The crystal structure of the title compound, viewed along the c axis, showing the molecules linked into dimers. H atoms not involved in intermolecular interactions (dashed lines) have been omitted for clarity.

## supplementary crystallographic information

### Comment

Photo-induced reactions between carbonyl groups acting as electron acceptors and substituted oxazoles as electron donors have been reported to proceed *via* [2+2] (Griesbeck *et al.*, 2003) or [4+4] photocycloaddition reactions (Zhang *et al.*, 2004). 1,3,4(2*H*)-Isoquinolinetrione derivatives have been used as carbonyl containing systems to take part in the photo-induced reactions with acetylenes (Zhang *et al.*, 2000). The reaction between 1,3,4(2*H*)-isoquinolinetrione and toluene gave the H-abstracted product (Suau & Villatoro, 1994). Hence the compounds containing functional groups with similar bond energy, for example, allyl or aldehyde, may give rise to photo-induced H-abstracted reaction. The crystal structure of *Z*-2-methyl-3'-phenyl-spiro[isoquinoline-4,2'-oxirane]-1,3-dione has been reported (Wang *et al.*, 2000). This paper reports the structure of the title compound, a typical H-abstracted product of the photoreaction between a carbonyl derivative and a thiazole.

In the title isoquinolinedione compound (Fig. 1), atom C9 is the chiral center. The piperidine ring (C1/N1/C2/C3/C8/C9) of the tetrahydroisoquinoline ring system adopts a half-boat conformation (Cremer & Pople, 1975) with puckering parameters of Q = 0.2975 (19) Å, θ = 70.7 (3)° and φ = 115.7 (4)° . The thiazol ring (C11/C12/S1/C13/N2) is essentially planar, with maximum deviation of 0.007 (2) Å at atom N2. The dihedral angle formed between the mean planes of the thiazol ring and the tetrahydroisoquinoline ring system is 34.49 (7)°. Bond lengths and angles are consistent with those in related isoquinoline-1,3-dione structures (Fun *et al.* 2010*a,b,c*; Zhang *et al.*, 2004).

In the crystal structure (Fig. 2), two inversion-related molecules are linked into dimers incorporating of *R*^2^_2_(9) hydrogen-bond ring motifs (Bernstein *et al.*, 1995) by O3—H1O3···N2 and C10—H10A···O1 hydrogen bonds (Table 1). These dimers are further interconnected by weak C14—H14B···*Cg*1 and C15—H15B···*Cg*2 interactions (Table 1) [*Cg*1 and *Cg*2 are the centroids of the  C3-C8 benzene ring and the thiazol ring, respectively].

### Experimental

The title compound was obtained in the reaction between 1,3,4(2*H*)-isoquinolinetrione (1 mmol, 189 mg) and 2,4,5-trimethyl thiazoles (6 mmol, 762 mg) in dry acetonitrile (50 ml) under 400 nm photo-irradiation. The compound was purified by flash column chromatography with ethyl acetate and petroleum ether (1:4, v:v). X-ray quality single crystals of the title compound were obtained through slow evaporation of solvents from a solution of acetone and petroleum ether (1:5, v:v).

### Refinement

All the H atoms were located from difference Fourier map [range of C—H = 0.93 (3) - 0.98 (3) Å] and allowed to refine freely.

### Crystal data

**Table:**

C_16_H_16_N_2_O_3_S	*F*(000) = 664
*M**_r_* = 316.37	*D*_x_ = 1.466 Mg m^−^^3^
Monoclinic, *P*2_1_/*c*	Cu *K*α radiation, λ = 1.54178 Å
Hall symbol: -P 2ybc	Cell parameters from 9047 reflections
*a* = 8.5793 (2) Å	θ = 5.1–67.1°
*b* = 10.4438 (2) Å	µ = 2.14 mm^−^^1^
*c* = 17.5496 (3) Å	*T* = 100 K
β = 114.304 (1)°	Block, colourless
*V* = 1433.09 (5) Å^3^	0.32 × 0.19 × 0.12 mm
*Z* = 4	

### Data collection

**Table:**

Bruker SMART APEX DUO CCD area-detector diffractometer	2343 independent reflections
Radiation source: fine-focus sealed tube	2311 reflections with *I* > 2σ(*I*)
none	*R*_int_ = 0.021
φ and ω scans	θ_max_ = 65.0°, θ_min_ = 5.1°
Absorption correction: multi-scan (*SADABS*; Bruker, 2009)	*h* = −10→8
*T*_min_ = 0.546, *T*_max_ = 0.782	*k* = −11→12
22926 measured reflections	*l* = −17→20

### Refinement

**Table:**

Refinement on *F*^2^	Secondary atom site location: difference Fourier map
Least-squares matrix: full	Hydrogen site location: inferred from neighbouring sites
*R*[*F*^2^ > 2σ(*F*^2^)] = 0.041	All H-atom parameters refined
*wR*(*F*^2^) = 0.135	*w* = 1/[σ^2^(*F*_o_^2^) + (0.0848*P*)^2^ + 0.3552*P*] where *P* = (*F*_o_^2^ + 2*F*_c_^2^)/3
*S* = 1.33	(Δ/σ)_max_ = 0.001
2343 reflections	Δρ_max_ = 0.87 e Å^−^^3^
264 parameters	Δρ_min_ = −1.01 e Å^−^^3^
0 restraints	Extinction correction: *SHELXTL* (Sheldrick, 2008), Fc^*^=kFc[1+0.001xFc^2^λ^3^/sin(2θ)]^-1/4^
Primary atom site location: structure-invariant direct methods	Extinction coefficient: 0.034 (2)

### Special details

**Table:**

Experimental. The crystal was placed in the cold stream of an Oxford Cryosystems Cobra open-flow nitrogen cryostat (Cosier & Glazer, 1986) operating at 100.0 (1)K.
Geometry. All esds (except the esd in the dihedral angle between two l.s. planes) are estimated using the full covariance matrix. The cell esds are taken into account individually in the estimation of esds in distances, angles and torsion angles; correlations between esds in cell parameters are only used when they are defined by crystal symmetry. An approximate (isotropic) treatment of cell esds is used for estimating esds involving l.s. planes.
Refinement. Refinement of F^2^ against ALL reflections. The weighted R-factor wR and goodness of fit S are based on F^2^, conventional R-factors R are based on F, with F set to zero for negative F^2^. The threshold expression of F^2^ > 2sigma(F^2^) is used only for calculating R-factors(gt) etc. and is not relevant to the choice of reflections for refinement. R-factors based on F^2^ are statistically about twice as large as those based on F, and R- factors based on ALL data will be even larger.

### Fractional atomic coordinates and isotropic or equivalent isotropic displacement parameters (Å^2^)

**Table:**

	*x*	*y*	*z*	*U*_iso_*/*U*_eq_	
S1	1.09845 (5)	0.83482 (4)	1.10440 (3)	0.0194 (3)	
O1	0.38500 (16)	0.80117 (12)	1.02541 (8)	0.0215 (4)	
O2	0.82233 (18)	0.52224 (13)	1.07705 (8)	0.0275 (4)	
O3	0.33049 (17)	0.82715 (12)	0.86199 (8)	0.0201 (4)	
N1	0.6048 (2)	0.66140 (13)	1.05324 (10)	0.0176 (4)	
N2	0.80660 (19)	0.91667 (14)	1.08986 (9)	0.0182 (4)	
C1	0.4850 (2)	0.74898 (16)	1.00286 (11)	0.0174 (4)	
C2	0.7103 (2)	0.58819 (16)	1.02692 (11)	0.0190 (4)	
C3	0.6699 (2)	0.58984 (16)	0.93626 (11)	0.0178 (4)	
C4	0.7471 (2)	0.49838 (17)	0.90499 (12)	0.0205 (4)	
C5	0.7076 (2)	0.49498 (17)	0.82010 (12)	0.0229 (5)	
C6	0.5912 (3)	0.58198 (18)	0.76658 (12)	0.0220 (4)	
C7	0.5162 (2)	0.67391 (17)	0.79782 (12)	0.0200 (4)	
C8	0.5556 (2)	0.67879 (16)	0.88301 (12)	0.0172 (4)	
C9	0.4934 (2)	0.78679 (17)	0.92028 (11)	0.0171 (4)	
C10	0.6204 (2)	0.90246 (16)	0.93867 (11)	0.0164 (4)	
C11	0.7918 (2)	0.88586 (16)	1.00992 (11)	0.0166 (4)	
C12	0.9358 (2)	0.84040 (15)	1.00461 (12)	0.0177 (4)	
C13	0.9603 (2)	0.89292 (17)	1.14552 (11)	0.0195 (4)	
C14	0.6343 (3)	0.65094 (18)	1.14136 (12)	0.0208 (5)	
C15	0.9659 (3)	0.8009 (2)	0.92990 (12)	0.0209 (4)	
C16	1.0194 (3)	0.9151 (2)	1.23738 (12)	0.0266 (5)	
H4A	0.824 (3)	0.439 (2)	0.9426 (14)	0.023 (5)*	
H5A	0.762 (3)	0.433 (2)	0.7991 (15)	0.032 (6)*	
H6A	0.563 (3)	0.581 (2)	0.7073 (15)	0.026 (6)*	
H7A	0.439 (3)	0.736 (2)	0.7631 (15)	0.027 (6)*	
H10A	0.564 (3)	0.975 (2)	0.9491 (13)	0.018 (5)*	
H10B	0.633 (3)	0.9172 (19)	0.8877 (14)	0.016 (5)*	
H14A	0.541 (4)	0.692 (3)	1.1484 (17)	0.040 (7)*	
H14B	0.634 (3)	0.561 (2)	1.1555 (14)	0.025 (5)*	
H14C	0.729 (4)	0.693 (3)	1.1756 (17)	0.033 (6)*	
H15A	0.988 (3)	0.711 (3)	0.9309 (17)	0.041 (7)*	
H15B	1.058 (3)	0.850 (2)	0.9259 (16)	0.033 (6)*	
H15C	0.869 (4)	0.820 (3)	0.8790 (19)	0.043 (7)*	
H16A	1.029 (4)	1.003 (3)	1.2454 (19)	0.056 (9)*	
H16B	0.939 (4)	0.885 (3)	1.2568 (18)	0.046 (7)*	
H16C	1.133 (4)	0.878 (3)	1.270 (2)	0.050 (8)*	
H1O3	0.297 (4)	0.889 (3)	0.8823 (19)	0.050 (8)*	

### Atomic displacement parameters (Å^2^)

**Table:**

	*U*^11^	*U*^22^	*U*^33^	*U*^12^	*U*^13^	*U*^23^
S1	0.0153 (4)	0.0216 (4)	0.0198 (4)	0.00071 (15)	0.0057 (2)	−0.00131 (15)
O1	0.0209 (7)	0.0208 (7)	0.0262 (7)	0.0007 (5)	0.0131 (6)	−0.0012 (5)
O2	0.0293 (8)	0.0307 (8)	0.0218 (7)	0.0109 (6)	0.0097 (6)	0.0053 (6)
O3	0.0146 (7)	0.0226 (7)	0.0198 (7)	0.0032 (5)	0.0037 (6)	0.0003 (5)
N1	0.0189 (9)	0.0167 (8)	0.0171 (8)	−0.0009 (6)	0.0075 (7)	−0.0003 (5)
N2	0.0184 (9)	0.0172 (8)	0.0187 (8)	−0.0018 (6)	0.0072 (6)	−0.0016 (6)
C1	0.0160 (9)	0.0146 (8)	0.0206 (9)	−0.0036 (7)	0.0067 (7)	−0.0025 (7)
C2	0.0188 (10)	0.0159 (9)	0.0223 (10)	−0.0005 (7)	0.0085 (8)	0.0004 (7)
C3	0.0173 (10)	0.0161 (9)	0.0209 (9)	−0.0035 (7)	0.0086 (7)	−0.0013 (7)
C4	0.0216 (10)	0.0171 (9)	0.0247 (9)	−0.0003 (7)	0.0112 (8)	0.0009 (7)
C5	0.0278 (11)	0.0181 (9)	0.0280 (10)	−0.0042 (8)	0.0167 (8)	−0.0054 (7)
C6	0.0273 (11)	0.0214 (9)	0.0197 (9)	−0.0079 (7)	0.0122 (8)	−0.0039 (7)
C7	0.0202 (11)	0.0187 (9)	0.0201 (10)	−0.0032 (7)	0.0073 (8)	0.0005 (7)
C8	0.0147 (10)	0.0166 (9)	0.0203 (9)	−0.0043 (6)	0.0073 (7)	−0.0020 (7)
C9	0.0133 (9)	0.0182 (9)	0.0176 (9)	−0.0002 (7)	0.0043 (7)	0.0000 (7)
C10	0.0173 (10)	0.0144 (9)	0.0175 (9)	0.0001 (7)	0.0071 (8)	0.0007 (7)
C11	0.0179 (10)	0.0130 (9)	0.0188 (9)	−0.0018 (6)	0.0075 (7)	0.0001 (6)
C12	0.0169 (10)	0.0145 (9)	0.0206 (10)	−0.0027 (6)	0.0066 (8)	−0.0002 (6)
C13	0.0180 (10)	0.0185 (9)	0.0221 (9)	−0.0017 (7)	0.0083 (7)	−0.0017 (7)
C14	0.0256 (12)	0.0205 (10)	0.0173 (10)	−0.0019 (8)	0.0098 (9)	0.0001 (7)
C15	0.0187 (10)	0.0234 (10)	0.0218 (10)	−0.0010 (8)	0.0095 (8)	−0.0012 (8)
C16	0.0220 (11)	0.0356 (12)	0.0202 (10)	0.0005 (9)	0.0065 (9)	−0.0045 (8)

### Geometric parameters (Å, °)

**Table:**

S1—C13	1.7317 (18)	C6—H6A	0.97 (2)
S1—C12	1.7337 (19)	C7—C8	1.391 (3)
O1—C1	1.212 (2)	C7—H7A	0.95 (3)
O2—C2	1.215 (2)	C8—C9	1.508 (2)
O3—C9	1.414 (2)	C9—C10	1.569 (2)
O3—H1O3	0.84 (3)	C10—C11	1.497 (2)
N1—C1	1.388 (2)	C10—H10A	0.95 (2)
N1—C2	1.400 (2)	C10—H10B	0.96 (2)
N1—C14	1.465 (2)	C11—C12	1.363 (3)
N2—C13	1.301 (2)	C12—C15	1.495 (3)
N2—C11	1.393 (2)	C13—C16	1.495 (3)
C1—C9	1.532 (2)	C14—H14A	0.96 (3)
C2—C3	1.482 (3)	C14—H14B	0.97 (3)
C3—C8	1.394 (3)	C14—H14C	0.90 (3)
C3—C4	1.397 (3)	C15—H15A	0.95 (3)
C4—C5	1.386 (3)	C15—H15B	0.97 (3)
C4—H4A	0.95 (2)	C15—H15C	0.96 (3)
C5—C6	1.390 (3)	C16—H16A	0.93 (3)
C5—H5A	0.96 (3)	C16—H16B	0.94 (3)
C6—C7	1.388 (3)	C16—H16C	0.98 (3)
			
C13—S1—C12	90.28 (9)	C1—C9—C10	107.64 (14)
C9—O3—H1O3	109 (2)	C11—C10—C9	116.27 (14)
C1—N1—C2	124.01 (15)	C11—C10—H10A	109.3 (13)
C1—N1—C14	118.86 (15)	C9—C10—H10A	106.8 (13)
C2—N1—C14	116.94 (15)	C11—C10—H10B	110.6 (13)
C13—N2—C11	110.88 (15)	C9—C10—H10B	105.4 (13)
O1—C1—N1	121.76 (16)	H10A—C10—H10B	108.0 (17)
O1—C1—C9	120.54 (16)	C12—C11—N2	116.10 (16)
N1—C1—C9	117.49 (15)	C12—C11—C10	126.12 (16)
O2—C2—N1	119.78 (16)	N2—C11—C10	117.76 (15)
O2—C2—C3	123.34 (16)	C11—C12—C15	130.18 (18)
N1—C2—C3	116.76 (15)	C11—C12—S1	108.55 (14)
C8—C3—C4	120.56 (17)	C15—C12—S1	121.27 (14)
C8—C3—C2	121.10 (16)	N2—C13—C16	124.69 (17)
C4—C3—C2	118.33 (16)	N2—C13—S1	114.19 (13)
C5—C4—C3	119.64 (17)	C16—C13—S1	121.11 (14)
C5—C4—H4A	121.7 (13)	N1—C14—H14A	108.1 (17)
C3—C4—H4A	118.7 (13)	N1—C14—H14B	108.9 (13)
C4—C5—C6	119.94 (17)	H14A—C14—H14B	108 (2)
C4—C5—H5A	119.3 (15)	N1—C14—H14C	112.7 (16)
C6—C5—H5A	120.7 (15)	H14A—C14—H14C	105 (2)
C7—C6—C5	120.40 (17)	H14B—C14—H14C	113 (2)
C7—C6—H6A	118.9 (13)	C12—C15—H15A	111.0 (16)
C5—C6—H6A	120.7 (13)	C12—C15—H15B	111.1 (15)
C6—C7—C8	120.17 (17)	H15A—C15—H15B	111 (2)
C6—C7—H7A	122.3 (14)	C12—C15—H15C	111.2 (17)
C8—C7—H7A	117.5 (14)	H15A—C15—H15C	108 (2)
C7—C8—C3	119.26 (16)	H15B—C15—H15C	104 (2)
C7—C8—C9	121.39 (16)	C13—C16—H16A	106.6 (19)
C3—C8—C9	118.99 (16)	C13—C16—H16B	111.3 (18)
O3—C9—C8	109.28 (15)	H16A—C16—H16B	108 (3)
O3—C9—C1	110.12 (14)	C13—C16—H16C	112.4 (18)
C8—C9—C1	112.37 (14)	H16A—C16—H16C	107 (3)
O3—C9—C10	108.37 (14)	H16B—C16—H16C	111 (3)
C8—C9—C10	108.97 (14)		
			
C2—N1—C1—O1	172.72 (16)	C3—C8—C9—C1	−30.6 (2)
C14—N1—C1—O1	−12.4 (2)	C7—C8—C9—C10	−84.4 (2)
C2—N1—C1—C9	−12.5 (2)	C3—C8—C9—C10	88.64 (19)
C14—N1—C1—C9	162.30 (16)	O1—C1—C9—O3	−30.9 (2)
C1—N1—C2—O2	173.39 (17)	N1—C1—C9—O3	154.29 (15)
C14—N1—C2—O2	−1.5 (2)	O1—C1—C9—C8	−152.96 (16)
C1—N1—C2—C3	−10.5 (2)	N1—C1—C9—C8	32.2 (2)
C14—N1—C2—C3	174.59 (15)	O1—C1—C9—C10	87.05 (19)
O2—C2—C3—C8	−171.67 (18)	N1—C1—C9—C10	−87.76 (18)
N1—C2—C3—C8	12.4 (2)	O3—C9—C10—C11	169.71 (14)
O2—C2—C3—C4	9.4 (3)	C8—C9—C10—C11	−71.47 (19)
N1—C2—C3—C4	−166.57 (16)	C1—C9—C10—C11	50.6 (2)
C8—C3—C4—C5	−0.9 (3)	C13—N2—C11—C12	−1.1 (2)
C2—C3—C4—C5	177.98 (16)	C13—N2—C11—C10	177.57 (15)
C3—C4—C5—C6	−0.3 (3)	C9—C10—C11—C12	92.3 (2)
C4—C5—C6—C7	1.1 (3)	C9—C10—C11—N2	−86.14 (19)
C5—C6—C7—C8	−0.7 (3)	N2—C11—C12—C15	−178.82 (17)
C6—C7—C8—C3	−0.5 (3)	C10—C11—C12—C15	2.7 (3)
C6—C7—C8—C9	172.49 (17)	N2—C11—C12—S1	0.32 (19)
C4—C3—C8—C7	1.4 (3)	C10—C11—C12—S1	−178.19 (14)
C2—C3—C8—C7	−177.54 (16)	C13—S1—C12—C11	0.35 (13)
C4—C3—C8—C9	−171.82 (16)	C13—S1—C12—C15	179.58 (15)
C2—C3—C8—C9	9.3 (2)	C11—N2—C13—C16	179.99 (17)
C7—C8—C9—O3	33.9 (2)	C11—N2—C13—S1	1.32 (19)
C3—C8—C9—O3	−153.11 (15)	C12—S1—C13—N2	−1.00 (14)
C7—C8—C9—C1	156.42 (16)	C12—S1—C13—C16	−179.72 (16)

### Hydrogen-bond geometry (Å, °)

**Table:**

Cg1 and Cg2 are the centroids of the C3–C8 benzene ring and the C11/C12/S1/C13/N2 thiazol ring, respectively.

**Table:**

*D*—H···*A*	*D*—H	H···*A*	*D*···*A*	*D*—H···*A*
O3—H1O3···N2^i^	0.84 (3)	2.35 (3)	3.174 (2)	167 (3)
C10—H10A···O1^i^	0.96 (2)	2.39 (2)	3.163 (2)	138 (2)
C14—H14B···Cg1^ii^	0.97 (3)	2.71 (3)	3.403 (2)	129 (2)
C15—H15B···Cg2^iii^	0.97 (3)	2.89 (2)	3.537 (2)	125.4 (18)

Symmetry codes: (i) −*x*+1, −*y*+2, −*z*+2; (ii) −*x*+1, −*y*+1, −*z*+2; (iii) −*x*+2, −*y*+2, −*z*+2.
